# MEDICAL MANAGEMENT OF PAIN IN GERIATRIC PATIENTS

**DOI:** 10.1093/geroni/igz038.1492

**Published:** 2019-11-08

**Authors:** Sonia Sehgal

**Affiliations:** University of California Irvine, Orange, California, United States

## Abstract

Pain and pain management is a growing concern for older adult patients and their health care providers. Upwards of 50% of older adults report having bothersome pain and 75% of these individuals report having pain in more than one location. Persistent pain is associated with decreased physical function, depression, isolation, and a decreased quality of life. Managing chronic pain in the setting of an opioid crisis requires special knowledge about the aging body so that medications can be used safely and effectively; and adjuvant therapies such as PT or acupuncture can be maximized. This segment of the symposium will review common challenges faced by primary care providers when managing chronic pain in older adults: the natural physiologic changes that occur in the aging body, co-morbid chronic diseases which can complicate pain syndromes, and assessing pain in cognitively impaired patients.

